# Association Between Trimethylamine N-oxide and Adverse Kidney Outcomes and Overall Mortality in Type 2 Diabetes Mellitus

**DOI:** 10.1210/clinem/dgae009

**Published:** 2024-01-24

**Authors:** Ping-Shaou Yu, Ping-Hsun Wu, Wei-Wen Hung, Ming-Yen Lin, Yen-Yi Zhen, Wei-Chun Hung, Jer-Ming Chang, Jong-Rung Tsai, Yi-Wen Chiu, Shang-Jyh Hwang, Yi-Chun Tsai

**Affiliations:** Division of Nephrology, Department of Internal Medicine, Kaohsiung Medical University Chung-Ho Memorial Hospital, Kaohsiung Medical University, Kaohsiung 807, Taiwan; Department of Internal Medicine, Kaohsiung Municipal Cijin Hospital, Kaohsiung 805, Taiwan; Graduate Institute of Medicine, College of Medicine, Kaohsiung Medical University, Kaohsiung 807, Taiwan; Division of Nephrology, Department of Internal Medicine, Kaohsiung Medical University Chung-Ho Memorial Hospital, Kaohsiung Medical University, Kaohsiung 807, Taiwan; Faculty of Medicine, College of Medicine, Kaohsiung Medical University, Kaohsiung 807, Taiwan; Division of Endocrinology and Metabolism, Department of Internal Medicine, Kaohsiung Medical University Chung-Ho Memorial Hospital, Kaohsiung Medical University, Kaohsiung 807, Taiwan; Division of Nephrology, Department of Internal Medicine, Kaohsiung Medical University Chung-Ho Memorial Hospital, Kaohsiung Medical University, Kaohsiung 807, Taiwan; Division of Nephrology, Department of Internal Medicine, Kaohsiung Medical University Chung-Ho Memorial Hospital, Kaohsiung Medical University, Kaohsiung 807, Taiwan; Department of Microbiology and Immunology, College of Medicine, Kaohsiung Medical University, Kaohsiung 807, Taiwan; Division of Nephrology, Department of Internal Medicine, Kaohsiung Medical University Chung-Ho Memorial Hospital, Kaohsiung Medical University, Kaohsiung 807, Taiwan; Graduate Institute of Medicine, College of Medicine, Kaohsiung Medical University, Kaohsiung 807, Taiwan; Department of Internal Medicine, Kaohsiung Municipal Cijin Hospital, Kaohsiung 805, Taiwan; Division of Nephrology, Department of Internal Medicine, Kaohsiung Medical University Chung-Ho Memorial Hospital, Kaohsiung Medical University, Kaohsiung 807, Taiwan; Faculty of Medicine, College of Medicine, Kaohsiung Medical University, Kaohsiung 807, Taiwan; Division of Nephrology, Department of Internal Medicine, Kaohsiung Medical University Chung-Ho Memorial Hospital, Kaohsiung Medical University, Kaohsiung 807, Taiwan; Graduate Institute of Medicine, College of Medicine, Kaohsiung Medical University, Kaohsiung 807, Taiwan; Division of Nephrology, Department of Internal Medicine, Kaohsiung Medical University Chung-Ho Memorial Hospital, Kaohsiung Medical University, Kaohsiung 807, Taiwan; Graduate Institute of Medicine, College of Medicine, Kaohsiung Medical University, Kaohsiung 807, Taiwan; Faculty of Medicine, College of Medicine, Kaohsiung Medical University, Kaohsiung 807, Taiwan; Division of General Medicine, Kaohsiung Medical University, Kaohsiung Medical University Chung-Ho Memorial Hospital, Kaohsiung 807, Taiwan

**Keywords:** prospective study, type 2 diabetes mellitus, trimethylamine-N-oxide, kidney outcome, all-cause mortality

## Abstract

**Context:**

Type 2 diabetes (T2D) is the major contributor to chronic kidney disease and end-stage kidney disease (ESKD). The influence of trimethylamine N-oxide (TMAO) on kidney outcomes in T2D remains unclear.

**Objective:**

To examine the association between fasting serum TMAO levels and adverse kidney outcomes in patients with T2D.

**Methods:**

Between October 2016 and June 2020, patients with T2D were recruited and monitored every 3 months until December 2021. Serum TMAO levels were assessed using liquid chromatography-mass spectrometry. The primary kidney outcomes were doubling of serum creatinine levels or progression to ESKD necessitating dialysis; the secondary kidney outcome was a rapid 30% decline in estimated glomerular filtration rate within 2 years. All-cause mortality was also evaluated.

**Results:**

Among the 440 enrolled patients with T2D, those in the highest serum TMAO tertile (≥0.88 μM) were older, had a longer diabetes duration, elevated blood urea nitrogen, and lower estimated glomerular filtration rate. Over a median follow-up period of 4 years, 26 patients (5.9%) had a doubling of serum creatinine level or progression to ESKD. After propensity score weighting, the patients in the highest serum TMAO tertile had a 6.45-fold increase in the risk of doubling of serum creatinine levels or progression to ESKD and 5.86-fold elevated risk of rapid decline in kidney function compared with those in the lowest tertile. Additionally, the stepwise increase in serum TMAO was associated with all-cause mortality.

**Conclusion:**

Patients with T2D with elevated circulating TMAO levels are at higher risk of doubling serum creatinine, progressing to ESKD, and mortality. TMAO is a potential biomarker for kidney function progression and mortality in patients with T2D.

Type 2 diabetes mellitus (T2D) poses a significant global health challenge and places a large economic burden on health care systems. According to the International Diabetes Federation, approximately 537 million adults aged 20 to 79 years were diagnosed with T2D worldwide in 2021, a number that is expected to increase to 783 million by 2045 (1). Moreover, T2D is the second leading cause of chronic kidney disease (CKD) and end-stage kidney disease (ESKD) (2).

Diabetic kidney disease (DKD) is a prevalent complication of diabetes. The pathophysiologic mechanisms of DKD are complex. Standard treatments including renin-angiotensin blockade, antidiabetic drugs, and sodium-glucose transporter 2 inhibitors are beneficial in reducing cardiovascular morbidity and slowing DKD progression; however, the risk of progressing to ESKD still persists (3). Novel biomarkers are needed for the early detection of kidney failure in patients with T2D to enable tailored treatments and improved outcomes.

Trimethylamine N-oxide (TMAO) is a gut microbiota-dependent metabolite that is primarily derived from the metabolism of dietary choline and carnitine and is found abundantly in red meat, eggs, and fish (4). Elevated TMAO levels have been associated with cardiovascular diseases, atherosclerosis, and metabolic disorders (5, 6). TMAO is predominantly excreted by the kidneys (6, 7) and has emerged as a potential biomarker and a contributing factor in the onset and progression of kidney disease through inflammation and oxidative stress (8-10), although few studies have investigated the impact of circulating TMAO levels on kidney progression and mortality in patients with T2D. Accordingly, the aim of this study was to elucidate the association between circulating TMAO levels and adverse kidney outcomes and all-cause mortality in patients with T2D.

## Materials and Methods

### Study Subjects

In this prospective study, we recruited patients with T2D at Kaohsiung Medical University Hospital (KMUH) on a consecutive basis from October 2016 to June 2020. During this period, all eligible patients with T2D attending outpatient departments were systematically and continuously invited to participate. T2D was defined as a medical history, blood glucose levels in accordance with the American Diabetes Association's criteria or the use of antidiabetic medications. All participants received instructions on an appropriate diet for diabetes. Kidney function was assessed by the estimated glomerular filtration rate (eGFR), as determined using the 2009 Chronic Kidney Disease Epidemiology Collaboration creatinine formula (11). We excluded patients with an eGFR < 15 mL/min/1.73 m², those with acute illnesses, and those who had used antimicrobials or probiotics within the 1 month before enrollment to diminish the effect of baseline kidney function and gut microbiome on the relationship between TMAO and adverse kidney outcomes. The protocol for this study was approved by the institutional review board of KMUH (KMUHIRB-G(II)-20160021), informed consent was obtained in written form from all study subjects, and all clinical investigations were conducted according to the principles expressed in the Declaration of Helsinki.

### Data Collection

Demographic characteristics, a history of currently or ever having smoked cigarettes or drunk alcohol, and clinical data from interviews and medical records were collected at enrollment. Hypertension was based on history or the use of antihypertensive drugs, ischemic heart disease was defined as a history of acute or chronic ischemic heart disease, or myocardial infarction, whereas heart failure was defined according to the previously published Framingham criteria (12). Body mass index was calculated as weight divided by height squared. Information regarding the use of antidiabetic drugs, insulin, statins, angiotensin-converting enzyme inhibitors, or angiotensin II receptor blockers before and after enrollment was obtained from medical records.

The patients were instructed to abstain from food for a minimum of 12 hours before the collection of blood and single-point urine samples the following morning for biochemical analysis. Serum creatinine was measured according to the compensated Jaffé (kinetic alkaline picrate) method using an autoanalyzer (Roche/Integra 400, Roche Diagnostics) and a calibrator that could be used in isotope-dilution mass spectrometry (13). Protein in urine was expressed as urinary albumin/creatinine ratio (UACR).

### Measurement of Fasting Serum TMAO

Twelve-hour fasting blood samples were processed and immediately stored at −80 °C for future analysis. TMAO is stable when stored at −80 °C for extended periods and can withstand multiple freeze-thaw cycles. TMAO was measured using the stable isotope dilution method, combined with high-performance liquid chromatography and online tandem mass spectrometry (14). The laboratory assay's coefficient of variation for TMAO fluctuated across different batches, with a maximum value of 5.8%.

### Kidney Outcomes and All-cause Mortality

The participants were monitored until the occurrence of specified kidney outcomes, mortality, final contact, or the conclusion of the observation period in December 2021. The patients were regularly assessed at outpatient clinics every 3 months to monitor their clinical status and measure eGFR. eGFR data obtained during hospitalization and episodes of acute kidney injury were excluded from the analysis to minimize the impact of acute illness on the assessment of the decline in renal function. The primary kidney outcomes were the doubling of serum creatinine levels or progression to ESKD; the secondary kidney outcome was defined as a 30% decline in eGFR within the first 2 years (15). Changes in eGFR were calculated as the percentage change between the first and last measurements (16). Cardiovascular mortality was defined as death resulting from ischemic heart disease, myocardial infarction, congestive heart failure, arrhythmia, or cerebrovascular accident. Information on all-cause mortality was obtained by direct contact with the participants and their families and was further supplemented by reviewing medical records and the databank of the National Mortality File.

### Statistical Analysis

Baseline characteristics were grouped by tertile TMAO level, with differences between continuous variables assessed using *t*-tests or Mann-Whitney *U* tests, and categorical variables using χ^2^ tests. Continuous variables with skewed distribution underwent log10 transformation to approximate a normal distribution before further analysis. We used the cumulative incidence competing risk method to account for competing risks, where death could preclude potential subsequent primary kidney outcomes (17, 18). Additionally, the Kaplan-Meier method was used for the analysis of all-cause mortality. We compared cumulative incidence functions using the Gray test, a technique similar to the log-rank test for Kaplan-Meier estimators (17). Propensity scores for TMAO tertile groups were estimated by a multinomial logistic regression model, ensuring balanced baseline covariates and reducing confounding factors across the 3 groups (19). Cox proportional hazards analysis with inverse probability of treatment weighting was used to examine the associations between TMAO level and primary kidney outcomes and all-cause mortality (20). Logistic regression models with inverse probability of treatment weighting were used to assess the association between serum TMAO level and 30% decline in eGFR within the initial 2 years (20). To evaluate the model classification performance, time-dependent receiver operating characteristic (ROC) curves were used to account for dynamic changes in disease status, offering a more precise evaluation of biomarker predictivity over time (21). This method was applied to appraise the prediction accuracy of TMAO, UACR, and eGFR for primary kidney outcomes at 1-, 3-, and 5-year intervals (21). Additionally, the Hosmer-Lemeshow test was used to assess the calibrations of TMAO, eGFR, and UACR separately in predicting the primary kidney outcomes (22, 23). We further analyzed factors that might modify the association between serum TMAO levels and the risk of progression to ESKD or doubling of serum creatinine, including sex (male/female), age (≥60 or <60 years), T2D duration (≥10 or <10 years), baseline eGFR (≥60 or <60 mL/min/1.73 m^2^), glycated hemoglobin (HbA1c; >7% or ≤7%), and baseline UACR (≥30 mg/g or <30 mg/g). Statistical analyses were executed using SPSS (version 20, IBM Corporation, Armonk, NY) and R statistical analysis software, version 4.2.2, with the “survival,” “survminer,” “dplyr,” and “cmprsk” packages for time-to-event evaluations and competing risk analysis, “timeROC” for estimation of time-dependent ROC curve, “twang” for propensity score estimating and weighting, and “rms” and “ggplot2” were used for modeling and visualization purposes (Foundation for Statistical Computing, Vienna, Austria; available at: http://www.R-project.org). All *P* values were 2-sided, with a significance threshold established at <.05.

## Results

### Characteristics of the Patients With T2D

Table 1 presents comparisons of baseline clinical characteristics, medication records, and laboratory parameters among participants stratified by tertiles of serum TMAO levels cut at 0.40 µM and 0.88 µM. The study population consisted of 440 patients with T2D and a mean age of 62.6 ± 10.9 years, of whom 55.0% were male. The median T2D duration was 9.0 years, and the prevalence rates of hypertension, heart failure, ischemic heart disease, and hyperlipidemia were 60.9%, 24.1%, 9.5%, and 78.2%, respectively. The median eGFR and HbA1c level were 79.8 mL/min/1.73 m^2^ and 7.0%, respectively. The patients in the highest tertile of serum TMAO levels were generally older, had a longer duration of T2D, and demonstrated higher usage of sulfonylurea and insulin compared with those in the lowest tertile. They also had higher baseline blood urea nitrogen and lower eGFR and hemoglobin levels than those in the lowest tertile.

**Table 1.** Characteristics of patients with T2D stratified by tertiles of baseline serum TMAO level

		Tertiles of TMAO, µM			
		T1	T2	T3	
	Entire cohort N = 440	(<0.40)	(≥0.40 to < 0.88)	(≥0.88)	
Variables		N = 146	N = 147	N = 147	*P*
Age (y)	62.6 ± 10.9	59.1 ± 12.0	63.8 ± 10.1	65.0 ± 9.8	<.001
Sex (male, %)	55.0	51.4	57.8	55.8	.53
Smoke (yes, %)	24.4	17.9	27.2	27.9	.087
Alcohol (yes, %)	20.7	16.6	24.5	21.1	.245
Hypertension (yes, %)	60.9	54.8	60.5	67.3	.09
Heart failure (yes, %)	24.1	19.2	27.9	25.2	.20
Ischemic heart disease (yes, %)	9.5	7.5	8.2	12.9	.23
Hyperlipidemia (yes, %)	78.2	76.0	81.6	76.9	.46
Duration of DM (y)	9.0 (4.0-15.0)	6.0 (3.0-12.0)	10.0 (5.0-15.0)	10.0 (5.0-18.0)	<.001
Body mass index (kg/m^2^)	26.2 (23.7-28.7)	26.4 (24.0-29.6)	25.4 (23.3-28.0)	26.6 (23.8-28.7)	.13
Dietary habit, %					.46
High animal- vs plant-based diet	14.9	12.8	12.8	19.0	
High plant- vs animal-based diet	36.1	33.6	38.5	36.5	
Balanced plant- and animal-based diet	48.9	53.6	48.7	44.4	
Medication					
Sulfonylurea (yes, %)	47.7	39.0	52.4	51.7	.04
DPP-4 inhibitor (yes, %)	65.7	63.7	63.3	70.1	.39
Metformin (yes, %)	83.6	81.5	87.1	82.3	.38
Pioglitazone (yes, %)	30.5	33.6	25.2	32.7	.23
Insulin (yes, %)	16.4	9.6	18.4	21.1	.02
SGLT2 inhibitor (yes, %)	2.0	0.7	3.4	2.0	.26
Statin (yes, %)	43.0	37.0	44.2	47.6	.17
ACEI/ARB (yes, %)	37.7	32.2	38.8	42.2	.20
Laboratory parameters					
eGFR (mL/min/1.73 m^2^)	79.8 ± 23.3	89.6 ± 18.4	80.2 ± 21.6	69.6 ± 25.0	<.001
eGFR ≥ 60	337 (76.6%)	136 (93.2%)	114 (77.6%)	87 (59.2%)	<.001
eGFR < 60	103 (23.4%)	10 (6.8%)	33 (22.4%)	60 (40.8%)	
Urinary ACR	17.5 (6.6-68.1)	13.8 (6.1-42.6)	16.4 (5.8-66.3)	23.2 (7.9-182.0)	.02
TMAO (µM)	0.62 (0.33-1.15)	0.24 (0.18-0.33)	0.61 (0.49-0.71)	1.56 (1.13-2.61)	<.001
Blood urea nitrogen (g/dL)	14.5 (11.8-18.5)	13.2 (10.5-15.5)	14.4 (11.4-18.5)	16.3 (13.6-22.9)	<.001
Hemoglobin (g/dL)	13.7 ± 1.7	14.0 ± 1.6	13.7 ± 1.49	13.3 ± 1.87	.001
ALT (IU/L)	33.8 ± 25.7	36.3 ± 25.8	32.5 ± 22.5	32.8 ± 28.7	.37
Cholesterol (mg/dL)	169 ± 39.3	170.4 ± 37.0	167.2 ± 42.1	169.8 ± 38.7	.76
Triglyceride (mg/dL)	122.0 (88-180.8)	125 (92-178.3)	122 (84-187)	118 (87 184)	.87
HbA1c (%)	7.0 (6.5-8.0)	6.9 (6.5-7.8)	7 (6.5-8)	7.2 (6.5-8.1)	.395

Data are expressed as numbers (percentages) for categorical variables and means ± SDs or medians (25th, 75th percentiles) for continuous variables as appropriate.

Abbreviations: ACEI, angiotensin converting enzyme inhibitors; ACR, albumin-creatinine ratio; ALT, alanine aminotransferase; ARB, angiotensin II receptor blockers; DM, diabetes mellitus; DPP-4, dipeptidyl peptidase 4; eGFR, estimated glomerular filtration rate; HbA1c, glycated hemoglobin; SGLT2, sodium–glucose cotransporter 2; T2D, type 2 diabetes mellitus; TMAO, trimethylamine-N-oxide.

### Serum TMAO Levels and Doubling of Serum Creatinine Level or Progression to ESKD

Over a mean follow-up period of 4.0 years, 26 patients (5.9%) reached a doubling of serum creatinine level or progression to ESKD (the primary kidney outcomes), 24 patients (5.5%) reached a doubling of serum creatinine level, 10 patients (2.3%) developed ESKD, and 15 patients (3.4%) died (Table 2). The patients in the highest tertile of serum TMAO had a higher incidence rate of primary kidney outcomes, including either doubling of serum creatinine level or ESKD, with an incidence rate of 35.1 per 1000 person-years compared with 9.1 and 3.6 per 1000 person-years in the patients in the middle and lowest tertiles, respectively.

**Table 2.** The studied outcomes and all-cause mortality of patients with T2D stratified by tertiles of baseline serum TMAO level

		Tertiles of TMAO, µM			*P*
		T1	T2	T3	
	Entire cohort N = 440	(<0.40)	(≥0.40 to <0.88)	(≥0.88)	
		N = 146	N = 147	N = 147	
Follow-up time (y)	4.0 (3.0-4.6)	4.1 (3.0-4.7)	4.1 (3.1-4.6)	4.0 (3.0-4.6)	.43
Numbers of serum creatinine measurement	10.8 ± 3.5	10.7 ± 3.6	10.7 ± 3.4	11.1 ± 3.6	.54
**Primary kidney outcomes**					
Incidence rate (per 1000 person-years)	15.9	3.6	9.1	35.1	<.001
ESKD or doubling of serum creatinine level (N, %)	26 (5.9)	2 (1.4)	5 (3.4)	19 (12.9)	<.001
Doubling of serum creatinine level (N, %)	24 (5.5)	2 (1.4)	5 (3.5)	17 (11.6)	<.001
End-stage kidney disease (N, %)	10 (2.3)	0 (0)	1 (0.7)	9 (6.1)	.001
**Secondary kidney outcomes**					
Incidence rate (per 1000 person-years)	13.8	3.6	5.5	33.4	<.001
30% decline of eGFR in the first 2 years (N, %)	22 (5.0)	2 (1.4)	3 (2.0)	17 (11.6)	<.001
**All-cause mortality**	15 (3.4)	1 (0.7)	4 (2.7)	10 (6.8)	.01

Data are expressed as numbers (percentages) for categorical variables and means ± SDs or medians (25th, 75th percentiles) for continuous variables as appropriate.

Abbreviations: DM, diabetes mellitus; eGFR, estimated glomerular filtration rate; ESKD, end-stage kidney disease; T2D, type 2 diabetes mellitus; TMAO, trimethylamine-N-oxide.

**Figure 1.**
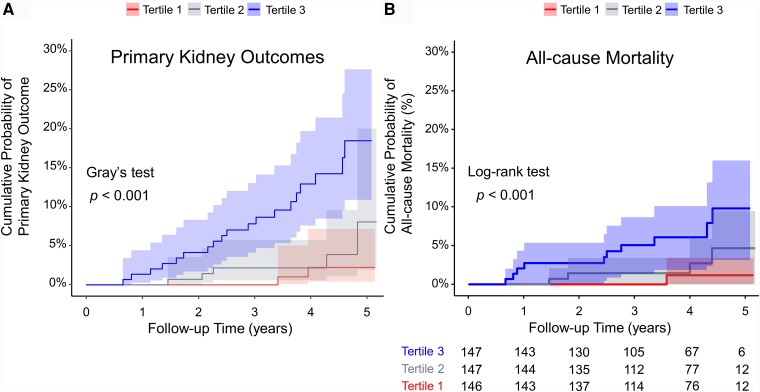
Cumulative probabilities of primary kidney outcome and all-cause mortality by TMAO tertile. (A) Cumulative probability curve of primary kidney outcomes (doubling of serum creatinine or dialysis) in patients with T2D, stratified by serum TMAO tertile, with all-cause mortality as a competing event. (B) One minus Kaplan-Meier plot: 5-year cumulative probability curve of all-cause mortality in patients with T2D, stratified by serum TMAO tertile. Abbreviations: T2D, type 2 diabetes; TMAO, trimethylamine-N-oxide.

Univariate analysis was performed to ascertain individual risk factors linked to the primary kidney outcomes. The results revealed positive correlations between the primary kidney outcomes and duration of T2D, body mass index, smoking, hypertension, UACR, log-transformed triglyceride, serum cholesterol, and log-transformed TMAO levels. Cumulative probability analysis showed a significantly elevated risk of primary kidney outcomes in the highest tertile compared with the middle and lowest tertiles (Gray test, *P* < .001; Fig. 1A and Supplementary Table S1) (24). After propensity score weighting, serum log-transformed TMAO was positively associated with primary kidney outcomes including doubling of serum creatinine Level or progression to ESKD (hazard ratio [HR], 5.32; 95% CI, 1.99-14.25). The patients in the highest tertile had a 6.45-fold (95% CI, 1.42-29.25) higher risk of reaching a primary outcome compared with those in the lowest tertile (Table 3).

**Table 3.** The association between serum TMAO level and the studied outcomes in patients with T2D

	Event N (%)	Crude HR*^a^*	PS-weighted HR*^b,c^*
		(95% CI)	(95% CI)
**ESKD or doubling of serum creatinine**	26 (5.9)	—	—
Log-transformed TMAO	—	7.58 (3.29-17.49)	5.32 (1.99-14.25)
Serum TMAO levels			
TMAO tertile 1 (<0.40 µM)	2 (1.4)	1.00 (Reference)	1.00 (Reference)
TMAO tertile 2 (≥0.40 to <0.88 µM)	5 (3.4)	2.60 (0.50-13.38)	2.40 (0.44-13.14)
TMAO tertile 3 (≥0.88 µM)	19 (12.9)	10.52 (2.45-45.21)	6.45 (1.42-29.25)
	Event N (%)	Crude HR*^a^*	PS-weighted HR*^b,c^*
		(95% CI)	(95% CI)
**Doubling of serum creatinine**	24 (5.5)	—	—
Log-transformed TMAO	—	7.60 (3.19-18.12)	5.31 (1.83-15.38)
Serum TMAO levels			
TMAO tertile 1 (<0.40 µM)	2 (1.4)	1.00 (Reference)	1.00 (Reference)
TMAO tertile 2 (≥0.40 to <0.88 µM)	5 (3.5)	2.60 (0.50-13.41)	2.40 (0.44-13.16)
TMAO tertile 3 (≥0.88 µM)	17 (11.6)	9.42 (2.17-40.85)	5.64 (1.23-25.79)
	Event N (%)	Crude OR*^d^*	PS-weighted OR*^b,c^*
		(95% CI)	(95% CI)
**30% decline in eGFR within 2 years**	22 (5.0)	**—**	**—**
Log-transformed TMAO		13.15 (4.57-37.83)	9.59 (2.94-31.19)
Serum TMAO levels			
TMAO tertile 1 (<0.40 µM)	2 (1.4)	1.00 (Reference)	1.00 (Reference)
TMAO tertile 2 (≥0.40 to <0.88 µM)	3 (2.0)	1.50 (0.25-9.11)	1.24 (0.18-8.56)
TMAO tertile 3 (≥0.88 µM)	17 (11.6)	9.42 (2.13-41.54)	5.86 (1.31-26.17)
	Event N (%)	Crude HR*^d^*	PS-weighted HR*^b,c^*
		(95% CI)	(95% CI)
**All-cause mortality**	15 (3.4)		
Log-transformed TMAO		4.24 (1.42-12.65)	4.58 (1.61-13.00)
Serum TMAO levels			
TMAO tertile 1 (<0.40 µM)	2 (1.4)	1.00 (Reference)	1.00 (Reference)
TMAO tertile 2 (≥0.40 to <0.88 µM)	3 (2.0)	4.14 (0.46-37.02)	4.00 (0.44-36.19)
TMAO tertile 3 (≥0.88 µM)	17 (11.6)	9.93 (1.27-77.55)	9.04 (1.13-72.66)

Abbreviations: ESKD, end stage kidney disease; HR, hazard ratio; OR, odds ratio; PS, propensity score; T2D, type 2 diabetes mellitus; TMAO, Trimethylamine-N-oxide.

^*a*^Crude hazard ratios, showing the direct association between the variable and the studied outcomes, were calculated using univariate Cox regression without adjustment for confounders.

^*b*^Propensity scores for TMAO tertile groups were estimated by a multinomial logistic regression model, ensuring balanced baseline covariates, and reducing confounding factors across the 3 groups. The covariates in the propensity score model reflect all baseline characteristics detailed in Table 1.

^*c*^Cox proportional hazards and logistic regression analysis with inverse probability of treatment weighting (IPTW) was used to examine associations between TMAO level and the studied outcomes.

^*d*^Crude odds ratio, showing the direct association between the variable and the studied outcome, was calculated using univariate logistic regression without adjustment for confounders.

### Model Performance of TMAO in Prediction of Doubling of Serum Creatinine Level or Progression to ESKD

**Figure 2.**
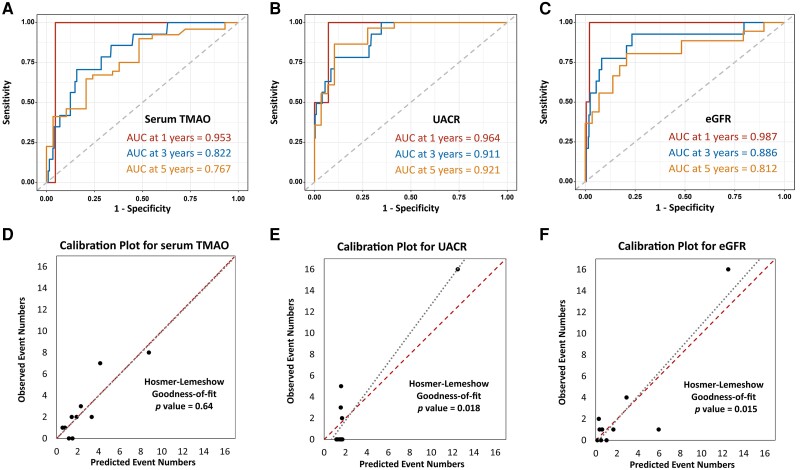
Time-dependent ROC curves and calibration plots for TMAO, UACR, and eGFR in predicting primary kidney outcome (doubling of serum creatinine or dialysis) in T2D. Time-dependent ROC curves for predicting 1-, 3-, and 5-year primary kidney outcomes (doubling of serum creatinine or dialysis) in patients with T2D by (A) serum TMAO, (B) UACR, and (C) eGFR. Calibration plot for predicting 1-, 3-, and 5-year primary kidney outcomes (doubling of serum creatinine or dialysis) in patients with T2D by (D) serum TMAO, (E) UACR, and (F) eGFR. Abbreviations: eGFR, estimated glomerular filtration rate; T2D, type 2 diabetes; TMAO, trimethylamine-N-oxide; UACR, urine albumin-to-creatinine ratio.

Time-dependent ROC analysis revealed that the area under the curve (AUC) for TMAO (0.953) was comparable to UACR (0.964) and eGFR (0.987) at 1 year, suggesting that serum TMAO had similar activity to predict kidney outcomes in T2D as UACR and eGFR at 1 year. At 3 and 5 years, the AUC of UACR was persistently higher than eGFR and serum TMAO, and the AUCs of serum TMAO and eGFR were similarly decreased over time (Fig. 2A–C). These findings indicated that TMAO's classification strength was not inferior with eGFR over time. The calibration of our prognostic models for serum TMAO, UACR, and eGFR was depicted graphically through scatter plots (Fig. 2D–2F), with predicted vs observed event numbers for primary kidney outcomes (Supplementary Tables S2A–C) (24). The TMAO-predicted events aligned closely with actual events, as indicated by the proximity of data points to the identity line (Fig. 2D). The nonsignificant Hosmer-Lemeshow test (*P* = .64) further supported that TMAO could adequately predict kidney outcome. Conversely, the calibration plots for UACR (Fig. 2E) and eGFR (Fig. 2F) identified a slight deviation from the expected number of primary kidney outcomes and failed Hosmer-Lemeshow test results (*P* = .018 for UACR and *P* = .015 for eGFR).

### Serum TMAO Levels and Rapid Decrease in Kidney Function

During the follow-up period, 22 (5.0%) patients had a decline in eGFR of ≥30% within the first 2 years. In addition, the patients in the highest tertile of serum TMAO had a higher incidence rate of kidney function decline (33.4 per 1000 person-years), compared with rates of 5.5 and 3.6 per 1000 person-years in the patients in the middle and lowest tertiles, respectively (Table 2). In univariate analysis, serum TMAO levels were positively correlated with a 30% decline in eGFR within the first 2 years. This association remained statistically significant after adjusting for confounders through propensity score weighting (Table 3). Serum log-transformed TMAO was positively associated with rapid decline in kidney function (HR, 9.59; 95% CI, 2.94-31.19). The patients in the highest tertile had a 5.86-fold (95% CI, 1.31-26.17) higher risk of reaching secondary outcome compared with those in the lowest tertile (Table 3).

### Subgroup Analysis of Serum TMAO Level and Doubling of Serum Creatinine Level or Progression to ESKD

We stratified the patients by sex (male/female), age (≥60 or <60 years), diabetes duration (≥10 or <10 years), baseline eGFR (≥60 or <60 mL/min/1.73 m^2^), HbA1c (> 7% or ≤7%), and UACR (≥30 mg/g or <30 mg/g) to examine the interaction effects of these factors on the relationship between circulating TMAO and primary kidney outcomes. The results revealed a significant interaction between TMAO level and sex (*P* = .018) in relation to the primary kidney outcomes (Supplementary Table S3) (24). There were no significant interaction effects between age, diabetes duration, baseline eGFR, UACR, or HbA1c on the association between circulating TMAO and primary kidney outcomes. These findings suggested a potential sex-specific relationship between TMAO and kidney health, with the male patients being more likely to reach a primary kidney outcome than the female patients.

### Serum TMAO and All-cause Mortality

The patients in the highest tertile of serum TMAO had a significantly higher all-cause mortality rate (5.9%, 13 deaths) compared with those in the middle (2.7%, 2 deaths) and lowest tertiles (0.7%, 1 death) (Table 2). The causes of mortality included cardiovascular events (4 cases), sepsis (5 cases), and cancer (6 cases). Besides, Kaplan-Meier analysis showed a greater cumulative probability of all-cause mortality in the highest TMAO tertile group (log-rank test, *P* < .001, Fig. 1B, and Supplementary Table S1) (24). After the propensity score weighting, serum log-transformed TMAO was found to be significantly correlated with all-cause mortality (HR, 4.58; 95% CI, 1.61-13.00). The patients in the highest tertile had an 9.04-fold increased risk of mortality (95% CI, 1.13-72.66) compared with their counterparts in the lowest tertile, indicating the potential link between elevated circulating TMAO level and increased risk of all-cause mortality.

## Discussion

This is the first prospective study to investigate the correlations between circulating TMAO levels and adverse kidney outcomes including doubling of serum creatinine level or progression to ESKD in T2D patients with proper glycemic control and preserved kidney function. After adjusting for established risk factors through propensity score weighting to balance the baseline covariates and reduce confounding across the 3 groups, the patients with a high circulating TMAO level had an elevated risk of reaching adverse kidney outcomes compared with those with a low circulating TMAO level. According to the results of reclassification ability and calibration, TMAO has the potential to be a reliable biomarker alongside eGFR and UACR in predicting adverse kidney outcomes. In addition, we further observed sex-specific variations in the impact of TMAO on kidney outcomes. The male patients with an elevated TMAO level had a higher risk of doubling serum creatinine or progression to ESKD, whereas the effect was less pronounced among the female patients.

Elevated TMAO levels have been demonstrated in patients with renal insufficiency (25, 26); meanwhile, previous cross-sectional studies have revealed an inverse relationship between TMAO and baseline kidney function (27, 28). Building on this evidence, we conducted this prospective study and found that elevated circulating TMAO levels were associated with an increased risk of doubling serum creatinine level or progression to ESKD requiring dialysis. This suggests that TMAO could serve as a potential biomarker for kidney function deterioration in patients with T2D. Furthermore, we found that the patients in the highest TMAO tertile had a higher risk of all-cause mortality compared with those in the lowest TMAO tertile. This finding is consistent with previous studies in which elevated circulating TMAO level was an independent predictor of overall mortality in patients with T2D (29, 30).

Existing research has mostly explored sex-based variations in TMAO level (31), focusing on potential causal factors such as the impact of hormones on the expression of the flavin-containing monooxygenase 3 gene (32). However, few studies have specifically investigated the adverse impact of TMAO on kidney function progression. In this study, we observed a sex-specific impact on the relationship between serum TMAO level and the risk of adverse kidney outcomes. Although there was no significant difference in TMAO concentration between the male and female patients, the impact of TMAO on kidney outcomes differed. The male patients with a TMAO level greater than the median had a significantly higher risk of adverse kidney outcomes, whereas the effect was less pronounced in the female patients. Further studies are needed to explore the factors contributing to the sex-specific differences in TMAO-related kidney outcomes.

Accumulating evidence has suggested a pathophysiologic role of TMAO in kidney injury (10, 33-35). TMAO has been found to induce kidney fibrosis by upregulating TGF-β1 in mice (10, 33). Fang et al demonstrated that TMAO activated NLR Family Pyrin Domain Containing 3 inflammasomes, leading to inflammation and the release of proinflammatory cytokines IL-1 and IL-18 in the kidneys (10); additionally, Hu et al found that decreasing TMAO production attenuated kidney injury through the inhibition of 3,3-dimethyl-1-butanol and microbiota depletion via antibiotic treatment in a murine CKD model (34). On the other hand, the gut microbiota has been shown to play a pivotal role in the TMAO pathway by metabolizing dietary precursors into trimethylamine within the human gut, which is then oxidized by the hepatic enzyme, flavin-containing monooxygenase, culminating in elevated TMAO levels (36). Nevertheless, research examining the link between TMAO, alterations in gut microbiota, and their specific effects on adverse kidney outcomes in T2D patients is limited, so further studies are required to clarify these potential associations (37).

Some limitations should be mentioned regarding the current study. We only assessed single baseline TMAO level and covariates such as HbA1c and low-density lipoprotein cholesterol, so the time-variant effects of TMAO and covariates might be underestimated in adverse kidney outcomes. Additionally, given the observational design of this study, we could not establish causality between TMAO level and kidney function progression. Although we collected information on the participants’ typical dietary habits, detailed data on diet content (energy intake and food components) were not available, potentially underestimating the influence of diet on host TMAO generation. However, we found no significant differences in dietary preferences among TMAO tertile groups, indicating that dietary factors might not have influenced our results.

In conclusion, our findings demonstrate that in patients with T2D with proper glycemic control and preserved baseline kidney function, an elevated serum TMAO level was associated with an increased risk of adverse kidney outcomes, as well as all-cause mortality. TMAO has the potential to be a reliable biomarker alongside eGFR and UACR in predicting adverse kidney outcomes in patients with T2D.

## Data Availability

The data that support the findings of this study are available from the corresponding author upon reasonable request.

## Abbreviations

AUC: area under the curve
CKD: chronic kidney disease
DKD: diabetic kidney disease
eGFR: estimated glomerular filtration rate
ESKD: end-stage kidney disease
HbA1c: glycated hemoglobin
HR: hazard ratio
KMUH: Kaohsiung Medical University Hospital
ROC: receiver operating characteristic
T2D: type 2 diabetes
TMAO: Trimethylamine N-oxide
UACR: urinary albumin/creatinine ratio

## References

[1] Sun H, Saeedi P, Karuranga S, et al IDF diabetes atlas: global, regional and country-level diabetes prevalence estimates for 2021 and projections for 2045. Diabetes Res Clin Pract. 2022;183:109119. 34879977 10.1016/j.diabres.2021.109119 PMC11057359

[2] Deng Y, Li N, Wu Y, et al Global, regional, and national burden of diabetes-related chronic kidney disease from 1990 to 2019. Front Endocrinol. 2021;12:672350. 10.3389/fendo.2021.672350 PMC8281340 34276558

[3] Chaudhuri A, Ghanim H, Arora P. Improving the residual risk of renal and cardiovascular outcomes in diabetic kidney disease: a review of pathophysiology, mechanisms, and evidence from recent trials. Diabetes Obes Metab. 2022;24(3):365‐376. 34779091 10.1111/dom.14601 PMC9300158

[4] Cho CE, Taesuwan S, Malysheva OV, et al Trimethylamine-N-oxide (TMAO) response to animal source foods varies among healthy young men and is influenced by their gut microbiota composition: a randomized controlled trial. Mol Nutr Food Res. 2017;61(1):1600324. 10.1002/mnfr.201600324 27377678

[5] Al-Rubaye H, Perfetti G, Kaski J-C. The role of microbiota in cardiovascular risk: focus on trimethylamine oxide. Curr Probl Cardiol. 2019;44(6):182‐196. 30482503 10.1016/j.cpcardiol.2018.06.005

[6] Li D, Lu Y, Yuan S, et al Gut microbiota–derived metabolite trimethylamine-N-oxide and multiple health outcomes: an umbrella review and updated meta-analysis. Am J Clin Nutr. 2022;116(1):230‐243. 35348578 10.1093/ajcn/nqac074 PMC9257469

[7] Cannon JA, McMurray JJV. Gut feelings about heart failure. *J* Am Coll Cardiol. 2014;64(18):1915‐1916. 10.1016/j.jacc.2014.04.088 25444146

[8] Tang WW, Wang Z, Kennedy DJ, et al Gut microbiota-dependent trimethylamine N-oxide (TMAO) pathway contributes to both development of renal insufficiency and mortality risk in chronic kidney disease. Circ Res. 2015;116(3):448‐455. 25599331 10.1161/CIRCRESAHA.116.305360 PMC4312512

[9] Tomlinson JA, Wheeler DC. The role of trimethylamine N-oxide as a mediator of cardiovascular complications in chronic kidney disease. Kidney Int. 2017;92(4):809‐815. 28807612 10.1016/j.kint.2017.03.053

[10] Fang Q, Zheng B, Liu N, et al Trimethylamine N-oxide exacerbates renal inflammation and fibrosis in rats with diabetic kidney disease. Front Physiol. 2021;12:682482. 34220546 10.3389/fphys.2021.682482 PMC8243655

[11] Levey AS, Stevens LA, Schmid CH, et al A new equation to estimate glomerular filtration rate. Ann Inter Med. 2009;150(9):604‐612. 10.7326/0003-4819-150-9-200905050-00006 PMC2763564 19414839

[12] McKee PA, Castelli WP, McNamara PM, Kannel WB. The natural history of congestive heart failure: the Framingham study. N Engl J Med. 1971;285(26):1441‐1446. 5122894 10.1056/NEJM197112232852601

[13] Vickery S, Stevens PE, Dalton RN, van Lente F, Lamb EJ. Does the ID-MS traceable MDRD equation work and is it suitable for use with compensated Jaffe and enzymatic creatinine assays? Nephrol Dial Transplant. 2006;21(9):2439‐2445. 16720592 10.1093/ndt/gfl249

[14] Le TT, Shafaei A, Genoni A, et al Development and validation of a simple LC-MS/MS method for the simultaneous quantitative determination of trimethylamine-N-oxide and branched chain amino acids in human serum. Anal Bioanal Chem. 2019;411(5):1019‐1028. 30552494 10.1007/s00216-018-1522-8

[15] Coresh J, Turin TC, Matsushita K, et al Decline in estimated glomerular filtration rate and subsequent risk of end-stage renal disease and mortality. JAMA. 2014;311(24):2518‐2531. 24892770 10.1001/jama.2014.6634 PMC4172342

[16] Levin A, Stevens PE, Bilous RW, et al Kidney disease: improving global outcomes (KDIGO) CKD work group. KDIGO 2012 clinical practice guideline for the evaluation and management of chronic kidney disease. Kidney Int Suppl. 2013;3(1):1‐150.

[17] Noordzij M, Leffondré K, van Stralen KJ, Zoccali C, Dekker FW, Jager KJ. When do we need competing risks methods for survival analysis in nephrology? Nephrol Dial Transplant. 2013;28(11):2670‐2677. 23975843 10.1093/ndt/gft355

[18] Austin PC, Lee DS, Fine JP. Introduction to the analysis of survival data in the presence of competing risks. Circulation. 2016;133(6):601‐609. 26858290 10.1161/CIRCULATIONAHA.115.017719 PMC4741409

[19] Ridgeway G, McCaffrey DF, Morral AR, et al Toolkit for Weighting and Analysis of Nonequivalent Groups: A Tutorial for the R TWANG Package. RAND Corporation; 2022.

[20] Chesnaye NC, Stel VS, Tripepi G, et al An introduction to inverse probability of treatment weighting in observational research. Clin Kidney J. 2021;15(1):14‐20. 35035932 10.1093/ckj/sfab158 PMC8757413

[21] Kamarudin AN, Cox T, Kolamunnage-Dona R. Time-dependent ROC curve analysis in medical research: current methods and applications. BMC Med Res Methodol. 2017;17(1):1‐19. 28388943 10.1186/s12874-017-0332-6 PMC5384160

[22] Tripepi G, Jager KJ, Dekker FW, Zoccali C. Statistical methods for the assessment of prognostic biomarkers (part II): calibration and re-classification. Nephrol Dial Transplant. 2010;25(5):1402‐1405. 20167948 10.1093/ndt/gfq046

[23] Tripepi G, Heinze G, Jager KJ, Stel VS, Dekker FW, Zoccali C. Risk prediction models. Nephrol Dial Transplant. 2013;28(8):1975‐1980. 23658248 10.1093/ndt/gft095

[24] Yu P, Wu P, Hung W, et al Supplementary file for “Association between trimethylamine N-oxide and adverse kidney outcomes, and overall mortality in Type 2 diabetes mellitus”. Date of deposit 22 December 2023. *Figshare*. Dataset. 10.6084/m9.figshare.24891993 PMC11244202 38267025

[25] Pelletier CC, Croyal M, Ene L, et al Elevation of trimethylamine-N-oxide in chronic kidney disease: contribution of decreased glomerular filtration rate. Toxins (Basel). 2019;11(11):635. 31683880 10.3390/toxins11110635 PMC6891811

[26] Zeng Y, Guo M, Fang X, et al Gut microbiota-derived trimethylamine N-oxide and kidney function: a systematic review and meta-analysis. Adv Nutr. 2021;12(4):1286‐1304. 33751019 10.1093/advances/nmab010 PMC8321840

[27] Al-Obaide MA, Singh R, Datta P, et al Gut microbiota-dependent trimethylamine-N-oxide and serum biomarkers in patients with T2DM and advanced CKD. J Clin Med. 2017;6(9):86. 28925931 10.3390/jcm6090086 PMC5615279

[28] Kalagi NA, Thota RN, Stojanovski E, Alburikan KA, Garg ML. Plasma trimethylamine N-oxide levels are associated with poor kidney function in people with type 2 diabetes. Nutrients. 2023;15(4):812. 36839170 10.3390/nu15040812 PMC9960644

[29] Kanitsoraphan C, Rattanawong P, Charoensri S, Senthong V. Trimethylamine N-oxide and risk of cardiovascular disease and mortality. Curr Nutr Rep. 2018;7(4):207‐213. 30362023 10.1007/s13668-018-0252-z

[30] Croyal M, Saulnier P-J, Aguesse A, et al Plasma trimethylamine N-oxide and risk of cardiovascular events in patients with type 2 diabetes. J Clin Endocrinol Metab. 2020;105(7):2371‐2380. 10.1210/clinem/dgaa188 32301490

[31] Manor O, Zubair N, Conomos MP, et al A multi-omic association study of trimethylamine N-oxide. Cell Rep. 2018;24(4):935‐946. 30044989 10.1016/j.celrep.2018.06.096

[32] Bennett BJ, de Aguiar Vallim TQ, Wang Z, et al Trimethylamine-N-oxide, a metabolite associated with atherosclerosis, exhibits complex genetic and dietary regulation. Cell Metab. 2013;17(1):49‐60. 23312283 10.1016/j.cmet.2012.12.011 PMC3771112

[33] Jiang S, Shui Y, Cui Y, et al Gut microbiota dependent trimethylamine N-oxide aggravates angiotensin II–induced hypertension. Redox Biol. 2021;46:102115. 34474396 10.1016/j.redox.2021.102115 PMC8408632

[34] Hu DY, Wu MY, Chen GQ, et al Metabolomics analysis of human plasma reveals decreased production of trimethylamine N-oxide retards the progression of chronic kidney disease. Br J Pharmacol. 2022;179(17):4344‐4359. 35428974 10.1111/bph.15856

[35] Zixin Y, Lulu C, Xiangchang Z, et al TMAO as a potential biomarker and therapeutic target for chronic kidney disease: a review. Front Pharmacol. 2022;13:929262. 36034781 10.3389/fphar.2022.929262 PMC9411716

[36] Tang WW, Wang Z, Levison BS, et al Intestinal microbial metabolism of phosphatidylcholine and cardiovascular risk. N Engl J Med. 2013;368(17):1575‐1584. 23614584 10.1056/NEJMoa1109400 PMC3701945

[37] Mosterd C, Kanbay M, van den Born B, Van Raalte D, Rampanelli E. Intestinal microbiota and diabetic kidney diseases: the role of microbiota and derived metabolites inmodulation of renal inflammation and disease progression. Best Pract Res Clin Endocrinol Metab. 2021;35(3):101484. 33546983 10.1016/j.beem.2021.101484

